# Dupilumab effectively and rapidly treats bullous pemphigoid by inhibiting the activities of multiple cell types

**DOI:** 10.3389/fimmu.2023.1194088

**Published:** 2023-07-27

**Authors:** Tianmeng Yan, Yinghan Xie, Yuhua Liu, Ying Shan, Xiaoyan Wu, Jing Wang, Ya-Gang Zuo, Zhenying Zhang

**Affiliations:** ^1^ Department of Dermatology, The First Affiliated Hospital of Jinan University, Guangzhou, China; ^2^ Department of Dermatology, The University of Hong Kong Shenzhen Hospital, Shenzhen, China; ^3^ Department of Dermatology, Peking Union Medical College Hospital, Beijing, China; ^4^ Department of Dermatology, The Eighth Affiliated Hospital of Sun Yat-sen University, Shenzhen, China

**Keywords:** bullous pemphigoid, dupilumab, eosinophil, TARC, ICOSL

## Abstract

**Background:**

Bullous pemphigoid (BP) is an autoimmune skin-blistering disease. Systemic corticosteroids remain the first line treatment for moderate-to-severe BP with the potential for severe adverse events. Dupilumab has emerged as an alternative option for BP patients.

**Objective:**

We evaluated the efficiency and safety of dupilumab on BP treatment and explored a mode of drug action in depth.

**Methods and results:**

A multicenter retrospective cohort included 20 BP patients who received dupilumab with or without systemic corticosteroid in dupilumab group, and 20 matched BP patients who received corticosteroid alone in conventional group. Serum samples were collected from 20 patients (10 from dupilumab group and 10 from conventional group) at baseline and week 4. Compared to systemic corticosteroid alone, dupilumab with or without systemic corticosteroid was similarly efficacious in clinical remission at week4 (complete remission plus partial remission: 100%) and week24 (complete remission plus partial remission:100%), but allowing significant decreases in the cumulative doses of corticosteroids with reducing the incidence of adverse events. However, dupilumab did not decrease BP180 antibody despite an obvious clinical improvement. Comparative plasma proteomic analysis performed before and after treatment in 3 BP patients from dupilumab group revealed that drug use was associated with 30 differentially expressed proteins, including 26 down-regulated and 4 up-regulated proteins. The former consisted of immune related proteins involved in T/B cell interactions (inducible T-cell co-stimulator ligand, ICOSL) and in the activation of eosinophils (PRG2), mast cells (S100A12), and complement (CR2). TARC and ICOSL levels correlated with BP severity in patients who received either dupilumab or conventional treatment.

**Conclusion:**

Dupilumab has similar efficacy in treating BP as conventional drugs, by inhibiting the activities of many types of immune cells and complement, and regulating the interactions between T and B cells.

## 1 Introduction

Bullous pemphigoid (BP) is an autoimmune skin disease and more frequently observed in the elderly. Its incidence is increasing as populations age (1). The first-line treatment for severe BP remains a systemic corticosteroid (2), the use of which is associated with increased risks of adverse events (AEs), especially in older patients with cardiovascular, cerebrovascular, gastrointestinal disease, or diabetes mellitus (3). Antibody-producing B cells, T cell subpopulations (e.g., Th2 cells, eosinophils, and mast cells), the complement system, and certain inflammatory factors are involved in the pathogenesis of BP (4–7). Antibodies or small molecular drugs targeting Th2 cells, eosinophils, mast cells, and complement have recently been used to treat BP (NCT04563923, NCT04035733, and NCT02226146) (8–10). Among them, dupilumab, an antibody targeting interleukin (IL)-4 and IL-13 produced by Th2 cells, afforded clear clinical improvement (9, 11). However, more clinical evidence is required.

Corticosteroids can inhibit the abnormal immune reactions of BP by reducing B cell proliferation, antibody production, eosinophil generation and survival, as well as the levels of cytokines produced by T cells. BP180 antibodies produced by B cells are the key pathogenic factor in BP development (12), whose level usually falls on corticosteroid treatment. Also, an increased eosinophil in plasma and skin is a prominent feature of BP (13). Toxic granule proteins (eosinophil cationic protein [ECP], major basic protein [MBP], or eosinophil peroxidase [EPO]) and a cytokine (matrix metalloproteinase 9 [MMP9])released by eosinophils promote keratinocyte separation (6). Eosinophil counts, which fall on corticosteroid treatment, correlate with BP activity and may be prognostic in certain patients (14, 15). In addition, the plasma level of thymus and activation regulated chemokine (TARC), an important Th2 chemotactic agent, correlated positively with the Bullous Pemphigoid Disease Area Index (BPDAI) score of patients on systemic corticosteroids (16). IL-4 and IL-13 produced by Th2 cells participate in eosinophil recruitment (17) and anti-BP180 antibody production (18). IL-4 modulates TARC expression when the PI3K pathway is directly activated by STAT6 (19). It is not yet clear whether the actions of dupilumab are similar to those of corticosteroids. We therefore performed a multicenter, retrospective cohort study to explore the efficacy and safety of dupilumab for BP patients. As a novel treatment option, we aimed to investigate the possible mechanism of dupilumab application in treating pemphigoid. Therefore, we further compared plasma proteomes profile of 3 BP patients from dupilumab group before and after dupilumab injections. The eosinophils, TARC, and inducible T-cell co-stimulator ligand (ICOSL) data efficiently revealed the responses of BP patients to both dupilumab and corticosteroids.

## 2 Methods

### 2.1 Patient information

This was a retrospective cohort study conducted from April 2020 to December 2021. BP patient data were collected from the Eighth Affiliated Hospital of Sun Yat-sen University, Peking Union Medical College Hospital, and the University of Hong Kong Shenzhen Hospital. All BP patients fulfilled the recognized diagnostic criteria (20) based on clinical presentation, histopathological findings, and direct or indirect immunofluorescence test results. Moderate-to-severe BP was defined as an affected body surface area (BSA) > 10% or a skin erosion/blister total score on the BPDAI > 15, a urticaria/erythema score on the BPDAI > 20 (21). According to the medical record, patients receiving dupilumab with or without systemic corticosteroid were included in the dupilumab group, while patients receiving systemic corticosteroid only were included in the conventional group. Patients without regular follow-up for 24 weeks were excluded. Medical records and photographs were reviewed. We retrieved demographic characteristics, any underlying diseases, the lesional features, histological and immunological findings, laboratory data, doppler vascular ultrasonography, bone densitometry, the drugs used, the responses at weeks 4 and 24, and AEs during a 24-week regimen. Initially missing data were obtained by calling patients or their guardians. This study was approved by the Ethics Committee Institutional Board of our local hospital. Written informed consent was obtained from all participants.

### 2.2 Dupilumab efficacy and safety in BP patients

#### 2.2.1 Primary outcomes

The clinical remission (complete + partial) rate at week 4 (20).

- Complete remission (CR): The absence of new or established lesions (blisters, eczematous lesions, urticarial plaques, or mucosal lesions) and pruritus.
- Partial remission (PR): The presence of transient new lesions that healed within 1 week.
- Mild new activity: Less than three lesions/month that did not heal within 1 week or an extension of established lesions or pruritus once weekly but less than daily in a patient who had achieved disease control; these lesions healed within 2 weeks.
- Relapse/flare: Appearance of at least three new lesions/month, or at least one large (> 10 cm in diameter) eczematous lesion, or urticarial plaques that did not heal within 1 week, or extension of established lesions or daily pruritus in a patient who had achieved disease control.

#### 2.2.2 Secondary outcomes

- The clinical remission (CR + PR) rates at weeks 12 and 24.
- AEs during the 24-week regimen. The severity of AE was classed according to Common Terminology Criteria for Adverse Events (CTCAE) v4.0.
- Cumulative doses of corticosteroids taken to week 4.
- Anti-BP180 antibody titers at weeks 0 and 4.
- Eosinophil percentages (EOS%) in peripheral blood at weeks 0 and 4.

### 2.3 Proteome profiling

Six plasma samples from three BP patients in the dupilumab group were collected before and after dupilumab injections. Patient 1 received both a dupilumab injection and a maintenance dose of corticosteroids after BP recurrence. Patients 2 and 3 were treated with dupilumab alone after primary BP attacks (details were summarized in supplemental patient information). Differentially expressed proteins (DEPs) were analyzed. Tandem mass tagging (TMT) coupled with liquid chromatography and tandem mass spectrometry (LC–MS/MS) were used to explore the proteomes. **Supplementary Methods 1** provides detailed information on plasma collection and purification, exosome depletion, peptide labeling, LC–MS/MS and bioinformatics analyses, and database searching.

### 2.4 Enzyme-linked immunosorbent assays

Serum BP180 IgG antibody, TARC, ICOSL and S100A12 levels in 40 plasma samples were quantitated by ELISAs in 20 BP patients (10 in the dupilumab group and 10in the conventional group) at baseline and at week 4 aftertreatment. All ELISAs followed the manufacturers’ instructions [BP180 (MBL, Nagoya, Japan), TARC (R&D Systems, Minneapolis, MN, USA), ICOSL (Cusabio Biotech Co., Wuhan, China), and S100A12 (Cusabio Biotech Co.)].

### 2.5 Statistical analysis

A Student’s t-test, Chi-Squared T test, Mann-Whitney U test, and Wilcoxon test, were performed as appropriate using SPSS ver. 26.0 software (IBM Corp., Armonk, NY, USA). A p-value < 0.05 was considered statistically significant.

## 3 Results

### 3.1 Dupilumab rapidly and effectively controlled BP without severe adverse events

Twenty patients with moderate-to-severe BP who received dupilumab alone or combined with conventional drugs and 20 age-, sex-, and disease severity-matched BP patients who received corticosteroid were recruited. All patients had been prescribed a background topical corticosteroid more than 1 week with poor clinical improvement. Among 40 patients, 22patients are newly diagnosed BP patients, and 18patients are recurrent ones. In conventional group, 10 of 20 patients received prior treatment with systemic corticosteroids before this recurrence, while in dupilumab group, 9 of 20 patients were treated with systemic corticosteroids or IVIG before. Notably, ten patients received dupilumab alone in the dupilumab group. Six patients in dupilumab group receiving systemic corticosteroid got poor outcomes after 1 week treatment and then received injections of dupilumab. The initial dupilumab dose was 600 mg followed by 300 mg weekly or every 1–4 weeks on 4 to 10 occasions, and the initial dosage of corticosteroid varied from 0.05mg/Kg to 2mg/Kg. By contrast, the initial dosage of corticosteroid in conventional group was varied from 0.2mg/Kg to 1mg/Kg prednisone depending on the disease severity and then was decreased at 2.5-10mg every 1-8 weeks in the follow-up. The baseline data are listed in **Table 1**. The details of dupilumab or prednisolone treatment protocol were listed in supplemental patient information.

**Table 1:** The baseline information of study population.

	Dupilumab group	Conventional group	All patients
Male	11	10	21
Female	9	10	19
Age (year)	73.4 ± 10.65	67.70 ± 11.76	70.55 ± 11.45
Duration before admission (month)	10.25 ± 16.91	9.95 ± 13.66	10.10 ± 15.18
EOS% within the normal range			
Yes	6	10	16
No	13*	9*	22
Underlying disease			
Cardiovascular disease	12	9	21
Neurologic disorders	7	4	11
Diabetes	6	3	9
others	7	4	11
Initial treatment	11	11	22
Recurrence treatment	9	9	18

*Both groups have one patient without eosinophilic data.

**Figure 1:**
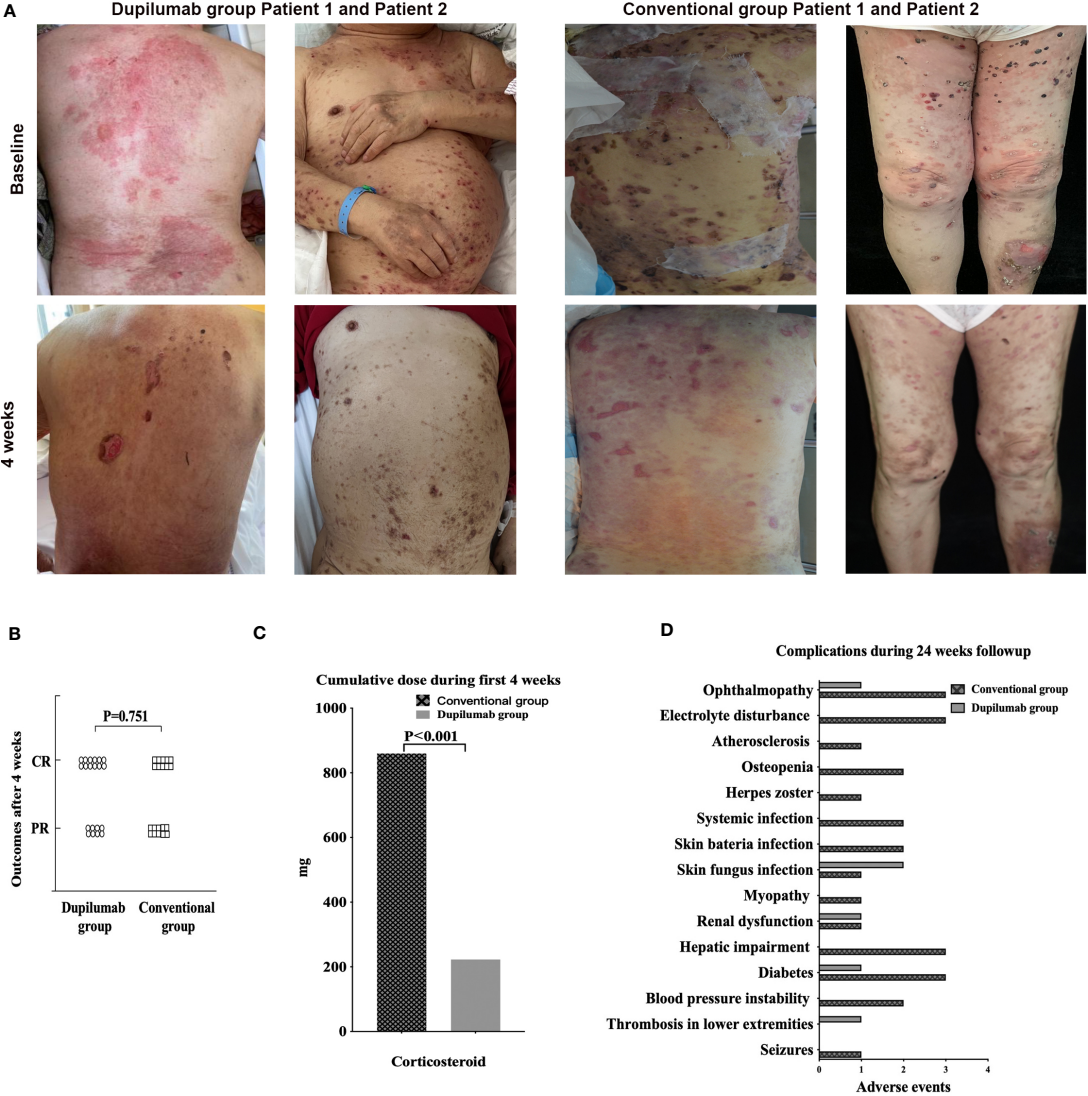
**(A)** Dupilumab effectively controlled BP without severe side effects. Clinical photographs taken at baseline and after 4 weeks from patients in dupilumab group and conventional group. **(B)** Primary outcomes of the dupilumab and conventional groups. In the former group, twelve patients achieved a CR and eight a PR; the figures for the conventional group were ten and ten. There was no significant difference between the two groups. **(C)** Cumulative doses of systemic corticosteroids at week 4, at which time BP was controlled in both the dupilumab and conventional groups. **(D)** Complications of BP patients treated with dupilumab or conventional drugs for 24 weeks. BP, bullous pemphigoid; CR, complete remission; PR, partial remission.

Of the 20 patients in the dupilumab group, twelve attained a CR with six on dupilumab alone. Eight patients achieved a PR with four on dupilumab alone (**Figure 1A**). In the conventional group, ten patients attained a CR and ten attained a PR (**Supplemental Figure 1**). The therapeutic efficacy did not differ between the two groups (p =0.751) (**Figure 1B**). Notably, dupilumab alone efficiently treated both initial episode and recurrences of pemphigoid. In the 20 patients on dupilumab, a CR was attained by 16/20, a PR by 4/20 at weeks 12 and a CR was attained by 17/20, a PR by 3/20 at 24 respectively. While in the conventional group, a CR was attained by 15/20 patients, a PR by 5/20 at week 12and a CR by 20/20 at week 24.

The cumulative corticosteroid dose decreased in the dupilumab group by week 4 when a CR or PR was attained (dupilumab group222.75 ± 454.48mg; conventional group 859.63 ± 379.32mg, p<0.01) (**Figure 1C**), and by week 24 (dupilumab group 835.25 ± 1373.31mg; conventional group 3189.33 ± 999.69mg, p<0.01). It is important to highlight that 12 of 20 in dupilumab group received dupilumab without corticosteroid. Further details are provided in supplemental patient information. The AE rate in the conventional group (26 AEs in fourteen patients) was significantly higher than in the dupilumab group (6 AEs in five patients) during the 24 weeks of treatment (**Figure 1D**). What’s more, AEs in the conventional group were more severe than that in the dupilumab group. According to CTCAE criteria, among 26AEs in the conventional group, twelve AEs were classified as grade 1, eleven were grade 2, and three were grade 3, while in the dupilumab group, two AEs were classified as grade 1, and four were grade 2. AEs were summarized in supplemental patient information.

### 3.2 The percentage of eosinophils but not the anti-BP180 IgG level decreased after dupilumab injection

**Figure 2:**
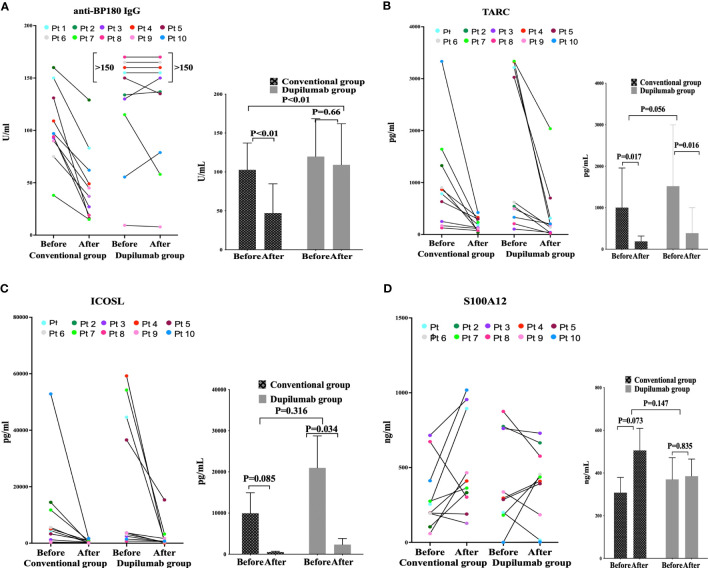
The enzyme-linked immunosorbent assay-based plasma levels of anti-BP180 IgG **(A)**, CCL17 **(B)**, ICOSL **(C)**, and S100A12 **(D)** before and after treatment in the conventional drug and dupilumab groups.

The percentage of eosinophils (EOS%) data at baseline and week4 were collected from 10 individuals in the dupilumab group and 10 in the conventional group whose serum anti-BP180 IgG antibody were detected. The EOS% decreased as BP became controlled after 4 weeks in both groups. In dupilumab group, the EOS% were15.25 ± 15.41% and 0.59 ± 0.95% before and after treatment, p = 0.013. In conventional group, the EOS% were 10.24 ± 11.50% and 0.28 ± 0.37% before and after treatment, respectively, p = 0.021. There EOS% level at baseline (p=0.430) and the reduction from baseline at week 4 (p=0.452) were similar between the groups. However, eight patients (five in the conventional group and three in the dupilumab group) evidenced normal eosinophil counts at baseline. The anti-BP180 antibody level decreased when a CR was attained at 4 weeks of treatment with conventional agents (before 102.70 ± 34.37 U/mL and after 48.60 ± 35.74U/mL, p <0.001) but not in the dupilumab group (before 119.73 ± 48.65 U/mL and after 116.66 ± 50.56 U/mL, p = 0.66) (**Figure 2A**). There was no statistically significant difference of anti-BP180 antibody level in the baseline between the groups (p=0.378). In the conventional group, the reduction from baseline of anti-BP180 antibody level was greater than in the dupilumab group at week 4 (p<0.001). Thus, dupilumab and corticosteroids have similarly effect in decreasing the EOS%.

### 3.3 Dupilumab inhibits the activities of multiple cell types

**Figure 3:**
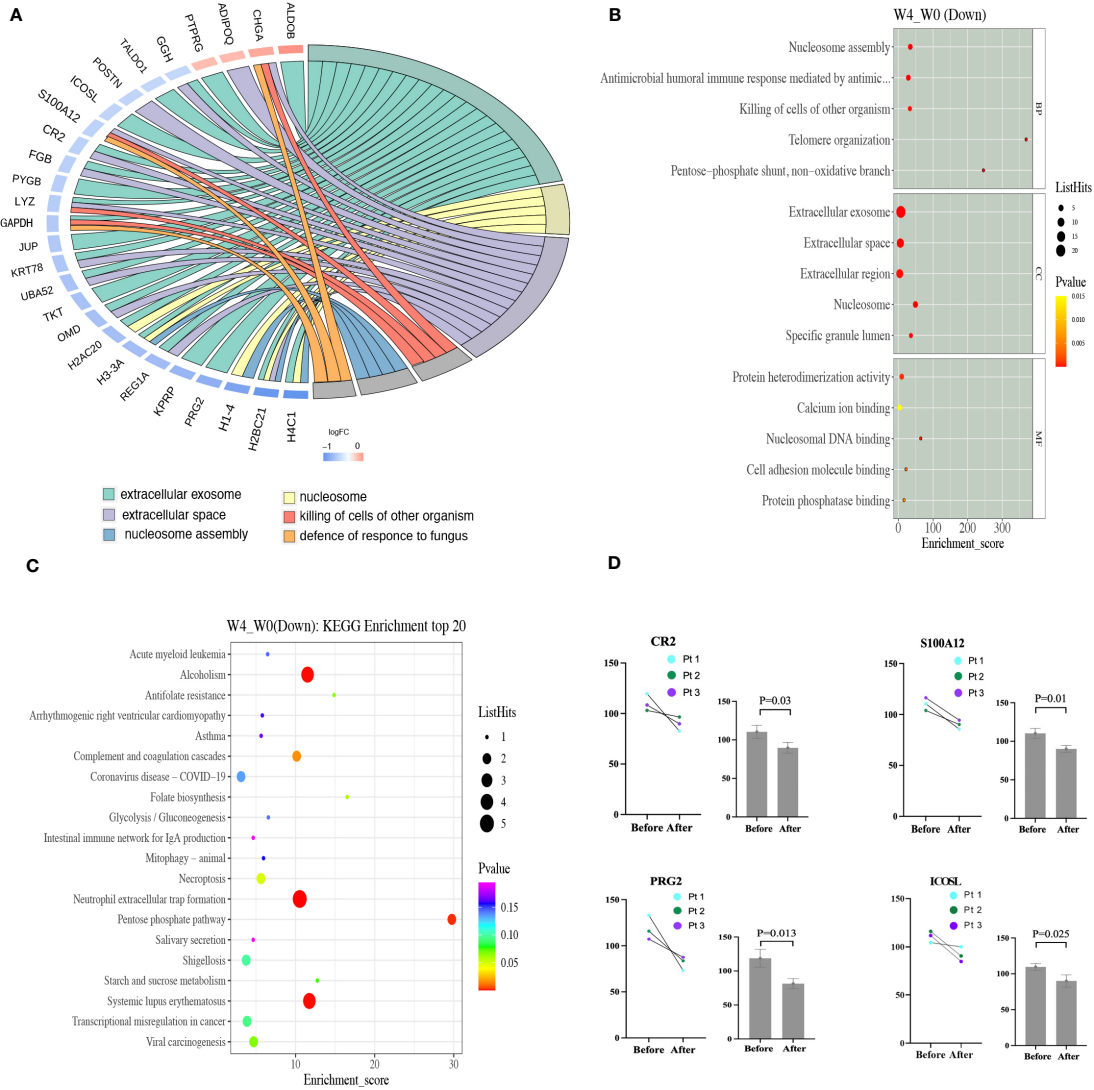
**(A)** The PPI network of DEPs based on STRING database analysis. Proteins up-regulated after dupilumab 4-week treatment are shown in orange and down-regulated proteins in blue (color of the outer circle). **(B)** The top 15 GO enrichment terms of the down-regulated DEPs are listed. The size of each bubble indicates the number of proteins it contains, and its color changes based on its p-value, with yellow representing the highest value and red representing the lowest. **(C)** Scatter plot of the top 20 pathways on KEGG enrichment of down-regulated DEPs. Larger bubbles indicate greater differences in the number of proteins, with their color transitioning from red to green, blue, and purple as the p-value increase and the difference becomes less significant. **(D)** The plasma levels of CR2, S100A12, PRG2, and ICOSL before and after 4-week dupilumab treatment in three patients as revealed by proteomic profiling. DEPs, different expressed proteins; GO, gene ontology.

Proteome profiling of six samples from 3 BP patients in dupilumab group (three samples before and three after treatment) revealed 879 unique proteins. Compared to the baseline, we detected 26 down-regulated and 4 up-regulated proteins at week 4 (Supplementary identified DEPs information). The roles played by several significantly down-regulated proteins, including ICOSL (before 109.65 and after 90.08, p = 0.025), CR2 (before 110.38 and after 89.67, p = 0.03), PRG2 (before 118.70 and after 81.40, p = 0.013), and S100A12 (before 110.31 and after 90.08, p = 0.01) are well understood (**Figure 3D**).

Gene ontology (Go) analysis was performed to comprehensively evaluate the plasma DEPs after dupilumab therapy. We explored molecular functions, the biological processes involved, and cellular components. Molecular function analysis revealed that some DEPs were associated with heterodimerization (H4C1, H2BC1, H3-3A, and H2AC20) or homodimerization (JUN, CR2, ADIPOQ, and TKT), calcium ion binding (H1-4, DSG1, TKT, and S100A12), and nucleosomal DNA binding (H3-3A and H1-4). Biological process analysis revealed that other DEPs were involved in nucleosome assembly (H2BC21, H1-4, and H3-3A), the antimicrobial humoral immune response (REG1A, GAPDH, S100A12, and H2BC21), and antibacterial defense (LYZ, S100A12, CHGA, PRG2, and GHV1-69D). Cellular component analysis revealed that some DEPs were enriched in extracellular exosomes (HABC21, PRG2, KPRP, REG1A, H3-3Q, H2AC20, OMD, TKT, UBA52, KRT78, JUP, GAPDH, LYZ, PYGB, FGB, CR2, ICOSL, TALDO1, GGH, PTPRJ, and ALDOB) which engage in intracellular molecule transportation (22), extracellular spaces (FGB, CR2, S100A12, POSTN, GGH, ADIPOQ, CHGA, H2BC21, REG1A, OMD, UBA52, KRT78, and LYZ), extracellular regions (S100A12, GGH, ADIPOQ, CHGA, H1-4, H3-3A, OMD, JUN, LYZ, PYGB, and FGB), the collagen-containing extracellular matrix (PRG2, OMD, FGB, POSTN, and ADIPOQ), and the lumen of a specific type of granule (JUN, LYZ, and GGH) (**Figure 3B**). Protein-protein interaction (PPI) analysis of all DEPs, performed with the aid of STRING, revealed that the top six categories were the extracellular exosome, nucleosome, extracellular space, killing of non-self-cells, nucleosome assembly, and defense against fungi (**Figure 3A**).

Kyoto Encyclopedia of Genes and Genomes (KEGG) pathway analysis disclosed that some DEPs affected neutrophil extracellular trap (NET) formation (H4C1, H2BC21, H3-3A, H2AC20, and FGB), the complement and coagulation cascades (CR2 and FGB), pentose phosphate metabolism (TKT, TALDO1, and ALDOB), and antifolate resistance (GGH) (**Figure 3C**). NET formation and the complement and coagulation cascades may play important roles in BP pathogenesis by promoting antibody production and blister formation (4, 23).

### 3.4 TARC and ICOSL are significantly down-regulated by dupilumab and corticosteroid

Compared to the pretreatment levels, the TARC levels decreased after treatment in both groups (dupilumab group: before 1,520.92 ± 1,477.20 pg/mL and after 386.86 ± 612.89 pg/mL, p = 0.016; conventional group: before 1,003.58 ± 953.50 pg/mL and after 190.22 ± 127.3 pg/mL, p = 0.017). The TARC levels at baseline and the reductions after treatment were similar in both groups (p = 0.056) (**Figure 2B**). Compared to before treatment, the ICOSL level decreased in both the conventional and dupilumab groups (dupilumab group: before 20.97 ± 24.55 ng/mL and after 2.35 ± 4.67 ng/mL, p = 0.034; conventional group: before 9.95 ± 15.78 ng/mL and after 0.544 ± 0.51 ng/mL, p = 0.085). The ICOSL levels at baseline and the reductions from baseline were similar in both groups (p = 0.316) (**Figure 2C**). The S100A12 levels before and after treatment did not differ between the two groups (dupilumab group: before 370.32 ± 321.04 ng/mL and after 385.49 ± 252.28 ng/mL, p = 0.835; conventional group: before 307.91 ± 225.15 ng/mL and after 505.83 ± 326.75 ng/mL, p = 0.073). The baseline S100A12 levels did not differ between the two groups (p = 0.147) (**Figure 2D**).

## 4 Discussion

In recent years, rituximab (an anti-CD20 antibody), dupilumab, and omalizumab (an anti-IgE antibody) have become alternative BP therapies (24). Dupilumab was efficacious, shortened the hospitalization time, allowed for corticosteroid tapering, and reduced complications (9, 11). Our results showed that dupilumab alone or combined with corticosteroid was competitive with corticosteroids in the treatment of BP with good tolerance, which were consistent with previous reports (25–30). Notably, dupilumab alone can control BP as early as week 4, reduced the cumulative doses of corticosteroids and fewer drug-associated AEs. What’s more, patients in dupilumab group had more health problems such as diabetes, neurologic disease, heart disease before treatment, but achieved less AEs than patients in conventional group. We recorded no conjunctivitis, parasitic infections, or eosinophilia; all are common during dupilumab therapy.

The pathogenesis of BP involves innate immunity [eosinophils (6), mast cells (31), and the complement system (4)], adaptive immunity [B (32), Th2 (5), Treg (33), and Tfh cells (34)], and complement-dependent and -independent pathways (4, 35–37). The complement-dependent pathway is initiated by antigen-antibody complexes in the basement membrane zone; these complexes activate complement (36–39). The complement-independent pathway is induced principally by antibodies and eosinophils, which results in blister formation regardless of complement activation (4, 13, 40–43). Plasma proteome profiling before and after dupilumab treatment revealed 26down-regulated proteins, such as ICOSL/B7H2, PRG2, S100A12, and CD21/CR2, which involved in T/B cell and B cell/complement interactions, eosinophil degranulation, and mast cell activation (44–47). ICOSL, a member of the B7 family of immune regulatory ligands, plays a vital role in the selection of high-affinity plasma cells, thus serving as a molecular linker of T and B cell interactions (44). CR2 recognizes complement 3 cleavage products bound to antigens and acts in conjunction with the B cell antigen receptor to lower the activation threshold and overcome B cell anergy (45). PRG2 participates in eosinophil activation, which is important in terms of BP pathogenesis (46). S100A12, a member of the S100 family of acidic calcium-binding proteins, may trigger mast cell degranulation and activation, in turn inducing the release of inflammatory mediators (47).

To investigate the effects of dupilumab on the activities of Th2 and mast cells, and T/B cell interactions, we measured the serum levels of TARC, S100A12, and ICOSL in 20 BP patients before and after treatment. The TARC and ICOSL levels decreased when BP was controlled. Surprisingly, the decreases occurred much earlier than the level of anti-BP180 antibody. Suzuki (16) found that the TARC level rather than the anti-BP180 level correlated with the BPDAI in BP patients on corticosteroids, consistent with our results. Similarly, there are decrease of TARC or eosinophils in peripheral blood and TARC, S100A12 or ICOS in skin lesions in atopic dermatitis patients after dupilumab treatment (48–52).

Bieber found that the S100A12 level fell in BP patients after 61 ± 40 weeks of therapy, as did the anti-BP180 level (53). In our plasma proteome profiling result from 3 patients in dupilumab group, S100A12 decreased after 4 weeks treatment. However, when we enlarged the samples to 10 patients, we found no decrease in S100A12 by week 4, which might indicate that mast cell activity differs between the early and late stages of BP remission.

The levels of eosinophils and anti-BP180 reflect disease severity in most BP patients (54, 55). In both groups, eosinophil levels clearly fell on entry into BP remission, in agreement with previous reports. However, dupilumab did not decrease the anti-BP180 antibody level despite the obvious clinical improvement, suggesting that corticosteroids and dupilumab act differently (56). Similar findings were reported in BP patients who received omalizumab. Balakirski et al. found no change in the level of anti-BP180 circulating autoantibodies after 6 months of omalizumab treatment (57). Yu et al. found that omalizumab slightly reduced the levels of anti-BP180 antibodies after disease remission (58). We speculate that dupilumab as well as omalizumab could not affect the production of IgG antibody directly but other pathways in pemphigoid disease.

In conclusion, dupilumab alone or combined with conventional drugs can control BP rapidly and safely over 24 weeks of treatment. Dupilumab may inhibit T/B cell interaction, and/or activate eosinophils, mast cells, neutrophils, and the complement system. Additional large-scale studies are required. Limited by sample size, missing data, and recall bias, further large-scale studies with prospective designs are warranted for the conclusion.

## Data availability statement

The original contributions presented in the study are included in the article/**Supplementary Material**, further inquiries can be directed to the corresponding authors.

## Ethics statement

The studies involving human participants were reviewed and approved by The university of Hong Kong-Shenzhen hospital. The patients/participants provided their written informed consent to participate in this study. Written informed consent was obtained from the individual(s) for the publication of any potentially identifiable images or data included in this article.

## Author contributions

TY analyzed the whole data and wrote the article. YX collected the information of BP patients in Peking Union Medical College Hospital and conducted the ELISA test. YL and XW collected the information of BP patients in The University of Hong Kong Shenzhen Hospital and The Eighth Affiliated hospital of Sun Yat-sen University and helped conducting the proteome profiling test. YS helped collected the information of BP patients in PUMCH. JW helped collecting the information of BP patients in Eighth Affiliated hospital of Sun Yat-sen University. ZZ and Y-GZ designed the research and revised the article. All authors contributed to the article and approved the submitted version.
