# Supplementary material for: Dupilumab effectively and rapidly treats bullous pemphigoid by inhibiting the activities of multiple cell types
Source: Front Immunol. 2023 Jul 27;14:1194088. doi: 10.3389/fimmu.2023.1194088 (PMC10421662; doi:10.3389/fimmu.2023.1194088)
Supplement: Supplementary file 1 [file DataSheet_1.docx]

Blood samples were centrifuged at 2000g for 15 min, with the supernatant collected and stored at -80 ◦C. Protein concentration was determined by BCA. For each sample, 50 μg of proteins were mixed with SDS to the same concentration and volume. Add DTT of into above protein solution to make the DTT final concentration about 5mM, and incubate at 55℃ for 30min. Then add corresponding volume of iodoacetamide so that the final concentration is about 10mM, and place in the dark for 15 min at room temperature. Then 6 times of the volume of precooled acetone in the above system to precipitate the protein, and place it at -20℃ for more than four hours or overnight.

After centrifugation, samples were washed with triethylammonium bicarbonate buffer (TEAB) and digested with trypsin-TPCK (Beijing life proteomic, Beijing, China) at 37 ◦C overnight. For TMT labelling, the lyophilized samples were resuspended in 50uL 100 mM TEAB. Eighty-eight microliter acetonitrile were added to TMT reagent vial at room temperature. The centrifuged reagents were dissolved for 5 min and mixed for centrifugation. Then 41 uL of the TMT label reagent was added to each sample for mixing. The tubes were incubated at room temperature for 1 h. finally, 8 uL of 5% hydroxylamine were added to each sample and incubated for 15 min to terminate the reaction. The labeling peptides solutions were lyophilized and stored at -80℃.

RP separation was performed on an 1100HPLC system (Agilent) using an Agilent Zorbax Extend RP column (5 um, 150 mm × 2.1 mm). Mobile phase A (2% acetonitrile in HPLC water) and B (90% acetonitrile in HPLC water) was used for RP gradient. The solvent gradient was set as follows: 0–8 min, 98% A; 8–8.01 min, 98% ~ 95% A; 8.01–48 min, 95% ~ 75% A; 48–60 min, 75–60% A; 60–60.01 min, 60–10% A; 60.01–70 min, 10% A; 70–70.01 min, 10–98% A; 70.01–75 min, 98% A. tryptic peptides were separated at a fluent flow rate of 300uL/min and monitored at 210 and 280 nm. Samples were collected for 8–60 min, and eluent was collected in centrifugal tube 1-15 every minute in turn. Samples were recycled in this order until the end of gradient. The separated peptides were lyophilized for mass spectrometry.

All analyses were performed by a Q-Exactive HF mass spectrometer (Thermo, USA) equipped with a Nanospray Flex source (Thermo, USA). Samples were loaded by a C18 column (2 cm × 100 μm,) and then separated by an C18 column (50 cm × 75 μm, Thermo, USA) on an Easy nanoLC-1200 system (Thermo, USA). The flow rate was 300 nL/min. Linear gradient was set as follows: 0~50 min, 2%~28% B; 50~60 min, 28%~42% B; 60~65 min, 42%~90% B; 65~75 min,90% B; Mobile phase A = 99.9% H2O/0.01% FA, Mobile B = 80% ACN/19.9%H2O/0.1% FA.

Full MS scans were acquired in the mass range of 350-1500 m/z with a mass resolution of 45000 the automatic gain control (AGC) target value was set as 3e6. The twenty most intense peaks in MS were fragmented with higher-energy collision dissociation with collision energy of 32. MS/MS spectra were obtained with a resolution of 15000 with an automatic gain control (AGC) target set as 2e6 and a max injection time of 80ms. The Q-E dynamic exclusion was set for 30 seconds.

Proteome Discover (v.2.4) was used to search all of the raw data thoroughly against the sample protein database. Database search was performed with Trypsin digestion specificity. Oxidation (M) and Acetyl (N-term) were considered as dynamic modifications in database searching. For protein quantification method, labeling method TMT6 was selected. A global false discovery rate (FDR) was set to 0.01 and proteins groups considered for quantification required at least 2 peptides.

GO term enrichment and KEGG pathway analysis DEPs were submitted to DAVID website (https://david.ncifcrf.gov/home.jsp) for functional annotation. Terms of biological process, cellular component and molecular function were analyzed according to the Gene Ontology (GO) database (http://amigo.geneontology.org) and functional pathway analysis were based on Kyoto Encyclopedia of Genes and Genomes (KEGG) database (http://www.kegg.jp/). Hypergeometric distribution test was used to determine the significance of differentially expressed protein enrichment in each GO term or KEGG pathway. The enrichment score was evaluated as follows: Enrichment score = (m/n) / (M/N) Here, N is the number of GO-annotated or KEGG-annotated proteins in all proteins, while n is the number of GO-annotated or KEGG-annotated proteins in DEPs in N. M is the number of proteins annotated to a particular GO term or KEGG pathway in all proteins while m is the number of proteins annotated to a particular GO term or KEGG pathway in DEPs.
