# Supplementary material for: Dupilumab effectively and rapidly treats bullous pemphigoid by inhibiting the activities of multiple cell types
Source: Front Immunol. 2023 Jul 27;14:1194088. doi: 10.3389/fimmu.2023.1194088 (PMC10421662; doi:10.3389/fimmu.2023.1194088)
Supplement: Supplementary file 2 [file Table_1.docx]

| Group | Age (year) | Sex | Duration (month) | Initial systemic treatment or Recurrence | Prior Medications | BPDAI before treatment | Current Treatment | BPDAI at week 4 | Response to treatment at week 4 | Response to treatment at week 12 | Response to treatment at week 24 | Cumulative corticosteroid dose during first 4 weeks (mg) | Cumulative corticosteroid dose during first 24 weeks (mg) | Adverse events during 24 weeks follow up | | | |
| --- | --- | --- | --- | --- | --- | --- | --- | --- | --- | --- | --- | --- | --- | --- | --- | --- | --- |
| Conventional group | | | | | | | | | | | | | | | | | |
| Patient1 | 67 | Female | 9 | R | MP | 143 | MP 40mg qd | 42 | PR | PR | CR | 1330 | 4690 | bacteria skin infection (grade 1) | hypoproteinemia（grade 1） | hyponatremia (grade 1) | |
| Patient2 | 69 | Female | 1 | I | none | 137 | MP 40mg qd | 26 | CR | CR | CR | 1330 | 3867.5 | corticosteroid induced myopathy (grade 2) | bacteria skin infections (grade2) |  | |
| Patient3 | 58 | Female | 2 | I | none | NA | MP 40mg qd | NA | CR | CR | CR | 1330 | 3710 | abnormal liver function (grade 1) | | | |
| Patient4 | 58 | Female | 2 | I | none | NA | MP 30mg qd | NA | CR | CR | CR | 805 | 3325 | urinary tract infection (grade 2) | pneumonia (grade 3) | osteopenia (grade1) | |
| Patient5 | 48 | Female | 2 | R | MP | NA | MP 48mg qd | NA | PR | CR | CR | 1645 | 5915 | abnormal liver function (grade 1) | hypocalcemia (grade 1) |  | |
| Patient6 | 73 | Female | 24 | R | MP | NA | MP 22mg qd | NA | CR | CR | CR | 757.5 | 2787.5 | intraocular hypertension (grade 2) | | | |
| Patient7 | 62 | Male | 3 | I | none | NA | MP 24mg qd | NA | CR | CR | CR | 840 | 2660 | osteopenia (grade 1) | | | |
| Patient8 | 63 | Male | 6 | R | MP | NA | MP 16mg qd | NA | CR | CR | CR | 560 | 2310 | abnormal liver function (grade 1) | tinea corporis (grade 1) | diabetes (grade 2) | atherosclerotic plaque of lower limbs (grade 1) |
| Patient9 | 66 | Male | 3 | I | none | NA | MP 24mg qd | NA | PR | CR | CR | 840 | 3220 | keratitis (grade 2) | conjunctivitis (grade 2) | | |
| Patient10 | 88 | Male | 60 | R | MP | NA | MP 24mg qd | NA | CR | CR | CR | 760 | 2580 | none |  |  |  |
| Patient11 | 79 | Female | 12 | R | MP | NA | Pre 30mg qd | NA | PR | PR | CR | 630 | 2844 | blood pressure instability （grade 3） |  |  |  |
| Patient12 | 79 | Female | 3 | R | Pre | NA | MP 16mg qd | NA | PR | PR | CR | 560 | 2520 | none |  |  |  |
| Patient13 | 63 | Male | 24 | R | MP | NA | MP 16mg qd | NA | CR | CR | CR | 560 | 2730 | none |  |  |  |
| Patient14 | 55 | Male | 5 | I | none | NA | MP24mg qd | NA | PR | CR | CR | 840 | 3640 | none |  |  |  |
| Patient15 | 88 | Male | 11 | R | MP | NA | MP 16mg qd | NA | CR | CR | CR | 560 | 2660 | unstable blood sugar (grade 2) |  |  |  |
| Patient16 | 50 | Male | 6 | I | none | NA | MP 16mg qd | NA | PR | PR | CR | 560 | 2660 | herpes zoster (grade 2) |  |  |  |
| Patient17 | 70 | Male | 12 | I | none | NA | MP 40mg qd | NA | PR | PR | CR | 1400 | 4760 | blood pressure instability （grade 3） |  |  |  |
| Patient18 | 87 | Female | 12 | I | none | NA | Pre 10mg qd | NA | PR | CR | CR | 280 | 1750 | diabetes (grade 2) | renal dysfunction (grade 2) | seizures (grade 1) |  |
| Patient19 | 66 | Female | 1 | I | none | NA | MP 12mg qd | NA | CR | CR | CR | 420 | 2310 | none |  |  |  |
| Patient20 | 65 | Male | 1 | I | none | NA | MP 40mg qd | NA | PR | CR | CR | 1185 | 2847.5 | none |  |  |  |
| Dupilumab group | | | | | | | | | | | | | | | | | |
| Patient1 | 79 | Female | 3 | R | none | 148 | Dup (600mg initial dose, 300mg weekly for 2 injections, every 2 weeks for 1 injection, total injections 4), MP 12mg qd | 11 | CR | CR | CR | 245 | 1120 | none |  |  |  |
| Patient2 | 77 | Male | 3 | I | none | 126.6 | Dup (600mg initial dose, 300mg weekly for 4 injections, every 2 weeks for 1 injection, total injections 6) | 39 | PR | CR | CR | 0 | 0 | none |  |  |  |
| Patient3 | 73 | Male | 2 | I | none | 125.2 | Dup (600mg initial dose, 300mg weekly for 3 injections, every 4 weeks for 5 injections, total injections 9) | 40.9 | PR | PR | PR | 0 | 0 | mucosa fungus infection (grade 2) |  |  |  |
| Patient4 | 74 | Female | 2 | I | none | 150 | Dup (600mg initial dose, 300mg every 2 week for 8 injections, total injections 9)，MP 8mg qd | NA | CR | CR | CR | 280 | 2240 | tinea pedis (grade 1) |  |  |  |
| Patient5 | 68 | Male | 4 | I | none | 134 | Dup (600mg weekly for 5 injections, total injections 10)，MP 16mg qd | NA | PR | CR | CR | 560 | 2520 | diabetes (grade 2) |  |  |  |
| Patient6 | 71 | Male | 2 | I | none | 134 | Dup (600mg initial dose, 300mg weekly for 8 injections, total injections 9) | NA | CR | CR | CR | 0 | 0 | none |  |  |  |
| Patient7 | 69 | Female | 72 | R | IVIG | 99 | Dup (600mg every 2 weeks for 4 injections,300mg every 2 week for 2 injections, total injections 10) | NA | PR | CR | CR | 0 | 0 | none |  |  |  |
| Patient8 | 52 | Female | 13 | R | MP | NA | Dup (600mg initial dose, 300mg every 2 week for 1 injection, total injections 2)，MP 40 mg bid | NA | CR | CR | CR | 1625 | 3830 | none |  |  |  |
| Patient9 | 86 | Male | 2 | I | none | 110 | Dup (600mg initial dose, 300mg every 2 weeks for 2 injections, every 4 weeks for 4 injections, total injections 7) | NA | CR | CR | CR | 0 | 0 | none |  |  |  |
| Patient10 | 70 | Female | 6 | I | none | 92 | Dup (600mg initial dose, 300mg every 2 weeks for 3 injections, total injections 4) | 13.6 | CR | CR | CR | 0 | 0 | none |  |  |  |
| Patient11 | 84 | Female | 36 | R | Pre | NA | Dup (600mg initial dose, 300mg q2w for 7 injections, total injections 8), MP 4mg qd | NA | PR | CR | CR | 140 | 840 | none |  |  |  |
| Patient12 | 88 | Male | 9 | I | none | NA | Dup (300mg q2w for 5 times, total injections 5) | NA | PR | PR | CR | 0 | 0 | renal dysfunction (grade 1) | thrombosis in lower extremities (grade 2) |  |  |
| Patient13 | 83 | Female | 6 | R | MP | NA | Dup (600mg initial dose, 300mg q2w for 2 times, total injections 3). MP 6 mg qd | NA | CR | CR | CR | 210 | 1260 | none |  |  |  |
| Patient14 | 70 | Female | 6 | R | MP | NA | Dup (600mg initial dose, 300mg q2w for 9 times, total injection | NA | CR | CR | CR | 0 | 0 | none |  |  |  |
| Patient15 | 67 | Male | 1 | I | none | NA | Dup (600mg initial dose, 300mg qw for 1time, total injections 2) | NA | PR | PR | PR | 0 | 0 | none |  |  |  |
| Patient16 | 48 | Female | 1 | I | none | NA | Dup 600mg,300mg qw for 1 time, q2w for 4 time.MP 40mg qd | NA | CR | CR | CR | 1325 | 4475 | keratitis (grade 2) |  |  |  |
| Patient17 | 84 | Male | 24 | R | MP | NA | Dup 600mg for 1 time), MP 2mg qd | NA | CR | CR | CR | 70 | 420 | none |  |  |  |
| Patient18 | 67 | Male | 1 | I | none | NA | Dup (600mg initial dose, 300mg q2w for 6 times, total injections 7) | NA | PR | PR | PR | 0 | 0 | none |  |  |  |
| Patient19 | 72 | Male | 6 | R | MP | 92 | Dup (600mg initial dose, 300mg every 2 weeks for 4 injections, total injections 5) | 20 | CR | CR | CR | 0 | 0 | none |  |  |  |
| Patient20 | 86 | Male | 6 | R | none | NA | Dup (600mg initial dose, 300mg every 2 weeks for 6 injections, total injections 7) | NA | CR | CR | CR | 0 | 0 | none |  |  |  |
| MP: methylprednisolone; I: initial systemic treatment; R: recurrence; CR: complement remission; PR: partial remission; IVIG: intravenous immunoglobulin  Dup (): dupilumab (regimen and total injections); | | | | | | | | | | | | | | | | | |
